# Does combining urine sediment examination to renal cell arrest and damage biomarkers improve prediction of progression and mortality of sepsis associated acute kidney injury?

**DOI:** 10.1186/s12882-025-04096-1

**Published:** 2025-04-17

**Authors:** Mohamed Mamdouh Elsayed, Ahmed Elsayed Eldeeb, Mona Moustafa Tahoun, Hala Saddik El-Wakil, Salah Said Naga

**Affiliations:** 1 Nephrology and Internal Medicine Department, Faculty of Medicine, Alexandria University, Alexandria, Egypt; 2 Clinical and Chemical Pathology Department, Faculty of Medicine, Alexandria University, Alexandria, Egypt

**Keywords:** Renal cell damage and arrest markers, TIMP-2, IGFBP7, KIM-1, Urine sediment examination, Sepsis associated acute kidney injury

## Abstract

**Background:**

Sepsis associated acute kidney injury (SA-AKI) among hospitalized patients is common with higher morbidity and mortality. There is a need to discover new methods that allow better prediction of its outcomes and prognosis. We aimed to evaluate if combining serial examination of urine sediment to renal cell damage (KIM-1) and arrest (TIMP-2, IGFBP7) biomarkers could improve the prediction of progression and mortality of SA-AKI.

**Methods:**

This prospective study enrolled 96 patients with stage 1 or 2 SA-AKI. Measuring of urinary TIMP-2, IGFBP7 and KIM-1 was done at time of AKI diagnosis and examination of urine sediment was performed by calculating Chawla score (CS) and Perazella score (PS) at days 1, 3 and 7. Main study outcomes included AKI progression to stage 3 and mortality.

**Results:**

Ninety-six patients were included in the study. 48% of them progressed to AKI stage 3 and 33.3% died. uTIMP2*IGFBP7 and uKIM-1 showed an area under the curve (AUC) of 0.837 and 0.657 respectively for predicting AKI progression and an AUC of 0.679 and 0.626 respectively for predicting mortality. Combining urine sediment examination at day 3 (P2 and C2) to uTIMP2*IGFBP7, uKIM-1 and both biomarkers significantly improved their prediction ability to an AUC of to 0.977, 0.951 and 0.979 respectively to predict AKI progression, and to an AUC of 0.807, 0.796 and 0.803 respectively to predict mortality.

**Conclusions:**

Combining urine sediment examination with renal cell damage and arrest biomarkers significantly improved their performance of predicting AKI progression and mortality in patients with SA-AKI.

**Clinical trials registration:**

ClinicalTrials.gov Identifier: NCT06064487. First registration date: 21/09/2023.

## Background

Acute kidney injury (AKI) is a frequent medical condition among hospitalized patients. AKI occurs in 31 to 65% of patients with septic shock and is linked to higher mortality and consumption of health care resources [1, 2].

A definition for sepsis-associated AKI (SA-AKI) was developed by the Acute Disease Quality Initiative (ADQI) 28 Workgroup [3] which combines sepsis, as defined by the Sepsis-3 criteria [4], with AKI, as defined by the Kidney Disease: Improving Global Outcomes (KDIGO) criteria [5], that occurs within a 7 days period after sepsis diagnosis. The features and outcomes of patients with SA-AKI differ from those of individuals with AKI of other etiologies [1]. Pathogenesis of SA-AKI has been linked to hemodynamic alterations, microcirculatory dysfunction, inflammation and metabolic reprogramming [6].

Finding and verifying novel biomarkers for predicting early AKI development and prognosis have garnered more attention recently. The FDA approved two biomarkers of renal cell cycle arrest, tissue inhibitor of metalloproteinase 2 (TIMP-2) and insulin-like growth factor binding protein 7 (IGFBP7), for assessing the possibility of severe AKI development. A risk score for stage 2–3 AKI occurrence has been issued by multiplying both biomarkers concentration in urine (i.e., u[TIMP-2]•[IGFBP7]) [7]. When renal cells are under stress or injury, they may release IGFBP7 and TIMP-2. This may support renal cells in maintaining energy balance and hindering more DNA damage [8].

Additionally, there are further novel biomarkers for renal injury, such as interleukin-18 (IL-18) and kidney injury molecule-1 (KIM-1), which indicate AKI inflammation and damage to the renal tubules. Both biomarkers demonstrated modest performance in predicting the course of AKI in the context of cardiac surgery and the ICU [9].

Urine sediment (U-Sed) examination is a well-established tool that has an important value in AKI. This procedure is noninvasive which could be performed manually or through an automated analyzer [10]. Still, manual U-Sed could provide much information which automatic analyzers cannot provide, mainly distinguishing dysmorphic from isomorphic red blood cells, recognizing renal tubular epithelial cells (RTECs), casts, and crystals. The identification of granular casts (GCs), RTECs and RTECs casts (RTECCs) suggests a diagnosis of acute tubular injury (ATI) [11]. The Perazella score (PS) and Chawla score (CS) were developed to help in standardization of U-Sed examination [12, 13]. So, in our study we aimed to evaluate if adding the U-Sed serial examination to renal cell arrest and damage biomarkers could improve the prediction of progression and mortality of SA-AKI.

## Methods

### Participants

Ninety-six patients with stage 1 or 2 SA-AKI admitted to Alexandria Main University Hospital between October 2023 and March 2024 were enrolled in the study. The KDIGO definition for AKI was used to diagnose AKI in our patients [5]. Sepsis was diagnosed according to Sepsis-3 criteria [4]. Sepsis should be diagnosed before or at the same time of AKI diagnosis. Patients were managed according to the Surviving Sepsis Campaign guidelines for treatment of septic patients. Patients with established chronic kidney disease with estimated glomerular filtration rate (eGFR) < 60 ml/min/1.73m^2^, aged < 18 years, hepatorenal syndrome, post renal obstruction, renal transplant recipients and pregnant females were excluded from the study. The trial was registered on Clinicaltrials.gov (NCT06064487).

### Methods & study outcomes

All patients underwent a thorough history taking, a comprehensive clinical examination, and an estimation of their urine output (UOP). The condition’s severity was evaluated by calculating the Acute Physiology and Chronic Health Evaluation II (APACHE II) score, the Sequential Organ Failure Assessment (SOFA) score and Charlson comorbidity index.

Laboratory parameters included serum creatinine, which was measured daily during hospital stay. For baseline serum creatinine, we used serum creatinine at hospital admission, the last available serum creatinine within the last three months or an estimated serum creatinine as per KDIGO guidelines in patients with no information about serum creatinine (a back formula was used to calculate baseline creatinine) [14]. Initial serum creatinine is the creatinine at time of AKI diagnosis.

Other laboratory parameters included blood urea, potassium, sodium, albumin, complete blood picture (CBC), lactate, C reactive protein (CRP), arterial blood gases, urinary albumin creatinine ratio (ACR) and urine analysis. The eGFR was calculated via the 2021 CKD-EPI creatinine equation.

Urinary samples for analysis of biomarkers were collected at time of AKI diagnosis. Urine collected was centrifuged at 2000–3000 rpm for 15 min and supernatants were frozen and stored at ≤ − 70 °C. Analysis was performed in the main laboratory of our hospital. Urinary TIMP2, IGFBP7 and KIM-1 were measured by ELISA kits (INNOVA BIOTECH CO. LIMITED, Daxing District, Beijing, China) according to the manufacturer’s instructions.

Urine sediment from a fresh urine sample was examined under a microscope, and the PS and CS were calculated at the time of AKI diagnosis (day 1), as well as on days 3 and 7. Urine was stored at room temperature, and an hour after it was collected, the U-Sed was examined. A 15 ml polypropylene conical tube containing 10 ml of urine was centrifuged at 800x g for 5 min. After pouring out the supernatant, the pellet was manually agitated in the 0.2 ml of supernatant that remained. A single drop was put on a slide, and an examination under the microscope was done using a 10x magnification eyepiece, and a 10x and 40x magnification objectives. The PS is calculated by counting the number of GCs in low-power field and the RTECs number in a high-power field with a score ranging from 0 to 4, whereas the CS is calculated by evaluating the percentage of GCs and RTECCs in low-power fields with a score ranging from 1 to 4. Two examiners independently assessed U-Sed under double-blind conditions.

Study outcomes included AKI progression (to KDIGO stage 3), duration of hospitalization and intensive care unit (ICU) stay, need for dialysis, mortality and renal functions at discharge and after 3 months.

### Statistical analysis

The package version 20.0 of the IBM SPSS software was used to analyze the data. (IBM Corp., Armonk, NY). Numbers and percentages were used to represent categorical data. Chi-square test was used to compare between two groups. Alternatively, Fisher Exact correction test was applied when the expected count was less than 5 in more than 20% of the cells. For continuous data, normality was tested by the Shapiro-Wilk test. The mean, standard deviation, range (minimum and maximum), median, and interquartile range were used to express the quantitative data. For normally distributed data, two groups were compared using the student t-test, and for non-normally distributed variables, two groups were compared using the Mann Whitney test. Kendall’s tau-b coefficient was used to correlate between two distributed not normally quantitative variables at least one of them was an ordinal variable. The diagnostic performance of the markers was assessed using a receiver operating characteristic curve (ROC); an area of greater than 50% indicates acceptable performance, while an area of almost 100% indicates the optimum performance. The results’ significance was assessed at the 5% level. Assuming that progression of mild/moderate AKI with sepsis to severe AKI occurred in 40%, so sample size is calculated to be at least 80 patients using OpenEpi software at confidence level 95% and power of study 80% [15].

## Results

### Baseline characteristics of patients

**Fig. 1:**
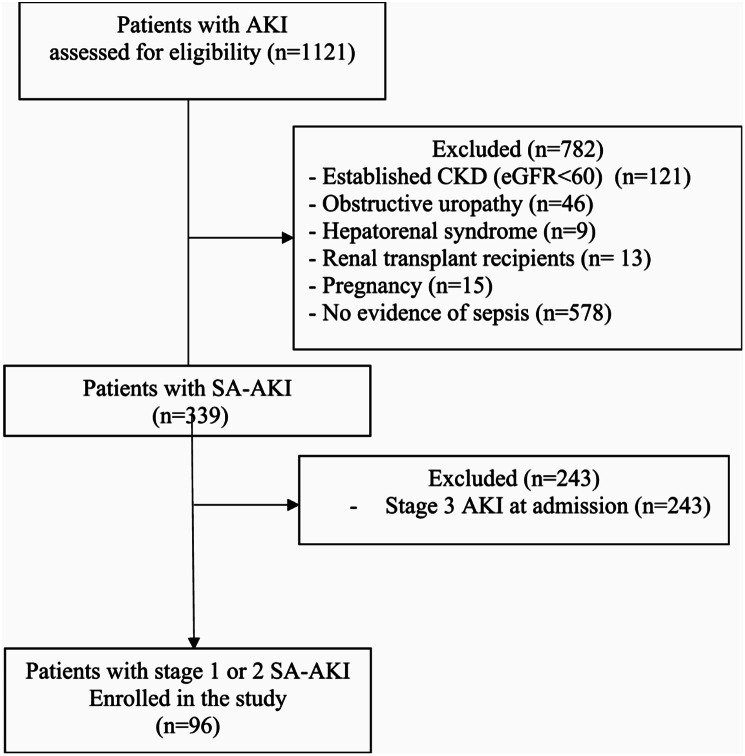
Patient flow chart

We assessed 1121 AKI patients to participate in our study. Of these, 1025 were excluded and 96 patients were enrolled (Fig. 1). The mean age of patients was 62.0 years, and 72.9% patients were females. Fifty-six patients (58.3%) had diabetes mellitus, and fifty-nine patients had hypertension (61.5%). At presentation, the mean serum creatinine was 1.9 mg/dl. There was no significant difference regarding baseline parameters between patients who progressed to AKI stage 3 and those who did not progress except for urine output at presentation which was significantly lower in AKI progressors (*p* = 0.015). Characteristics of patients at baseline are presented in Table 1.

**Table 1:** Baseline characteristics of patients

	Total (*n* = 96)	Progression		*p*
		No (*n* = 50)	Yes (*n* = 46)	
**Age (years)**	62.0 (53.0–67.0)	65.0 (55.0–69.0)	62.0 (50.0–66.0)	0.119
**Gender**				
Male	26 (27.1%)	17 (34.0%)	9 (19.6%)	0.112
Female	70 (72.9%)	33 (66.0%)	37 (80.4%)	
**Comorbidities**				
Diabetes mellitus	56 (58.3%)	25 (50.0%)	31 (67.4%)	0.084
Hypertension	59 (61.5%)	30 (60.0%)	29 (63.0%)	0.760
Charlson comorbidity index	5.0 (3.0–6.0)	4.50 (3.0–6.0)	5.0 (4.0–7.0)	0.202
**Source of infection**				
Chest	34 (35.4%)	16 (32.0%)	18 (39.1%)	0.490
UTI	48 (50.0%)	28 (56.0%)	20 (43.5%)	
Chest and UTI	9 (9.4%)	3 (6.0%)	6 (13.0%)	
Cellulitis	5 (5.2%)	3 (6.0%)	2 (4.3%)	
**Condition severity**				
SOFA score	4.0 (3.0–6.0)	4.0 (2.0–6.0)	5.0 (3.0–6.0)	0.063
APACHE II score	25.0 (12.0–30.0)	25.0 (12.0–33.0)	25.0 (12.0–30.0)	0.917
**UOP** (ml/kg/h)	0.27 (0.21–0.40)	0.30 (0.23–0.46)	0.23 (0.20–0.33)	0.015^*^
**Renal function**				
Baseline Creatinine (mg/dl)	0.90 (0.80–1.0)	0.90 (0.80–1.0)	0.90 (0.80–1.0)	0.940
Initial Creatinine (mg/dl)	1.90 (1.60–2.30)	1.80 (1.50–2.30)	2.0 (1.80–2.40)	0.204
Baseline eGFR (mL/min/1.73 m^2^)	75.0 (68.0–75.0)	75.0 (70.0–77.90)	75.0 (63.0–75.0)	0.402
Initial eGFR (mL/min/1.73 m^2^)	32.20 (25.0–45.0)	35.70 (25.0–45.0)	30.0 (25.90–45.0)	0.912
Urinary ACR (mg/g Cr)	211.0 (88.0–394.5)	210.0 (88.0–368.0)	246.0 (88.0–432.0)	0.397
Urea (mg/dl)	105.0 (84.0–140.0)	107.0 (80.0–140.0)	102.0 (92.0–149.0)	0.574
**Other laboratory parameters**				
Hemoglobin (g/dl)	9.90 (8.30–10.95)	9.0 (8.0–11.30)	10.30 (8.90–10.90)	0.749
WBCs	14.50(11.30–17.80)	12.80(10.90–17.80)	15.05(11.40–17.80)	0.266
Platelets	224.0(148.5–334.0)	221.0(139.0–297.0)	226.0(178.0–334.0)	0.153
Sodium (mmol/L)	132.0(129.0–135.0)	133.0(131.0–137.0)	131.0(128.0–135.0)	0.082
Potassium (mmol/L)	4.80 (4.20–5.30)	5.0 (4.20–5.40)	4.40 (4.20–5.20)	0.247
Albumin (g/dl)	2.90 (2.80–3.20)	2.90 (2.50–3.20)	3.05 (2.90–3.20)	0.051
pH	7.34 (7.28–7.38)	7.34 (7.31–7.39)	7.34 (7.28–7.38)	0.636
Lactate (mmol/L)	3.70 (3.0–4.20)	3.70 (2.70–4.10)	3.70 (3.40–4.20)	0.174
Bicarbonate (mmol/L)	17.0 (14.0–19.70)	16.80 (14.0–20.0)	17.0 (15.0–18.0)	0.971
CRP (mg/dl)	140.0 (82.0–172.5)	118.0 (82.0–171.0)	160.0 (98.0–173.0)	0.516
Procalcitonin (mg/L)	6.20 (3.10–8.50)	5.29 (2.67–7.95)	6.89 (3.12–9.85)	0.125

Qualitative data were described using number and percent while not normally distributed data was expressed in Median (IQR)

*p*: p value for comparing between AKI progressors and non-progressors

*: Statistically significant at *p* ≤ 0.05

ACR: albumin to creatinine ratio, APACHE II score: Acute Physiology and Chronic Health Evaluation II score, CRP: C reactive protein, eGFR: estimated glomerular filtration rate, SOFA score: Sequential Organ Failure Assessment score, UOP: urine output, UTI: urinary tract infection

### AKI outcomes

Forty-six patients (47.9%) progressed to AKI stage 3, while 50 patients (52.1%) did not progress. AKI progressors (to stage 3) had a significantly higher mortality, need for dialysis, ICU admission, need for mechanical ventilation and vasopressor drugs, longer hospital stay, higher serum creatinine and lower eGFR at discharge and after 3 months in comparison to patients with no AKI progression (Table 2).

**Table 2:** AKI outcomes in study patients

	Total (*n* = 96)	Progression		*p*
		No (*n* = 50)	Yes (*n* = 46)	
**Mortality**	32 (33.3%)	3 (6.0%)	29 (63.0%)	< 0.001^*^
**Need for Dialysis**	24 (25.0%)	0 (0.0%)	24 (52.2%)	< 0.001^*^
**Days of hospital admission**	8.0 (5.0–9.0)	6.0 (4.0–9.0)	8.0 (7.0–10.0)	< 0.001^*^
**ICU course**				
ICU admission	50 (52.1%)	19 (38.0%)	31 (67.4%)	0.004^*^
Days of ICU admission	5.62 ± 2.14	4.95 ± 2.68	6.03 ± 1.64	0.123
Need for ventilation	42 (43.8%)	11 (22.0%)	31 (67.4%)	< 0.001^*^
Need for vasopressor	34 (35.4%)	11 (22.0%)	23 (50.0%)	0.004^*^
**Renal functions**				
Creatinine at discharge (mg/dl)	1.40 (1.0–1.80)	1.10 (1.0–1.45)	2.10 (1.80–2.40)	< 0.001^*^
Creatinine after 3 months (mg/dl)	1.0 (1.0–1.30)	1.0 (0.90–1.10)	1.40 (1.30–1.50)	< 0.001^*^
eGFR at discharge (mL/min/1.73 m^2^)	49.75 (35.35–67.0)	60.0 (47.0–69.50)	33.10 (20.10–35.0)	< 0.001^*^
eGFR after 3 months (mL/min/1.73 m^2^)	61.0 (52.0–71.0)	65.0 (60.70–72.0)	45.10 (43.0–51.0)	< 0.001^*^

Qualitative data were described using number and percent while normally quantitative data was expressed in Mean ± SD and not normally distributed data was expressed in Median (IQR)

*p*: p value for comparing between AKI progressors and non-progressors

*: Statistically significant at *p* ≤ 0.05

ICU: intensive care unit

### Urine sediment scores, renal cell arrest and damage biomarkers

Urinary TIMP-2*IGFBP-7 and KIM-1 measured at time of diagnosis of AKI were significantly higher in patients who progressed to AKI stage 3 compared to non-progressors (*p* < 0.001 and 0.008) respectively. There was no statistically significant difference between AKI progressors and non-progressors in Perazella score at the time of diagnosis of AKI (P1) (*p* = 0.125), however, there was a significant difference in Chawla score (C1) (*p* = 0.018). Additionally, there was a significant difference between AKI progressors and non-progressors in Chawla and Perazella scores at day 3 (C2, P2) (*p* < 0.001 and < 0.001) respectively and day 7 (C3, P3) (*p* < 0.001 and < 0.001) respectively (Table 3).

**Table 3:** Renal cell arrest and damage markers and urine sediment scores in study patients

	Total	Progression		*p*
		No	Yes	
**IGFBP-7 × TIMP-2** (ng/ml)^2^/1000)	0.48 (0.33–0.75)	0.34 (0.16–0.46)	0.66 (0.49–2.17)	< 0.001^*^
**KIM-1** (pg/ml)	1310 (1070–1545)	1235 (875–1505)	1437.5 (1150–1690)	0.008^*^
**P1**	**(** ***n*** ** = 96)**	**(** ***n*** ** = 50)**	**(** ***n*** ** = 46)**	
0	27 (28.1%)	16 (32.0%)	11 (23.9%)	0.125
1	44 (45.8%)	26 (52.0%)	18 (39.1%)	
2	22 (22.9%)	7 (14.0%)	15 (32.6%)	
3 or more	3 (3.1%)	1 (2.0%)	2 (4.3%)	
**P2**	**(** ***n*** ** = 96)**	**(** ***n*** ** = 50)**	**(** ***n*** ** = 46)**	
0	17 (17.7%)	17 (34.0%)	0 (0.0%)	< 0.001^*^
1	22 (22.9%)	19 (38.0%)	3 (6.5%)	
2	27 (28.1%)	11 (22.0%)	16 (34.8%)	
3 or more	30 (31.3%)	3 (6.0%)	27 (58.7%)	
**P3**	**(** ***n*** ** = 70)**	**(** ***n*** ** = 35)**	**(** ***n*** ** = 35)**	
0	19 (27.1%)	18 (51.4%)	1 (2.9%)	< 0.001^*^
1	12 (17.1%)	11 (31.4%)	1 (2.9%)	
2	14 (20.0%)	5 (14.3%)	9 (25.7%)	
3 or more	25 (35.7%)	1 (2.9%)	24 (68.6%)	
**C1**	**(** ***n*** ** = 96)**	**(** ***n*** ** = 50)**	**(** ***n*** ** = 46)**	
1	47 (49.0%)	31 (62.0%)	16 (34.8%)	0.018^*^
2	47 (49.0%)	18 (36.0%)	29 (63.0%)	
3 or more	2 (2.1%)	1 (2.0%)	1 (2.2%)	
**C2**	**(** ***n*** ** = 96)**	**(** ***n*** ** = 50)**	**(** ***n*** ** = 46)**	
1	34 (35.4%)	33 (66.0%)	1 (2.2%)	< 0.001^*^
2	31 (32.3%)	16 (32.0%)	15 (32.6%)	
3 or more	31 (32.3%)	1 (2.0%)	30 (65.2%)	
**C3**	**(** ***n*** ** = 70)**	**(** ***n*** ** = 35)**	**(** ***n*** ** = 35)**	
1	31 (44.3%)	30 (85.7%)	1 (2.9%)	< 0.001^*^
2	15 (21.4%)	4 (11.4%)	11 (31.4%)	
3 or more	24 (34.3%)	1 (2.9%)	23 (65.7%)	

Qualitative data were described using number and percent and not normally distributed data was expressed in Median (IQR)

*p*: p value for comparing between the two studied groups

*: Statistically significant at *p* ≤ 0.05

P1, P2 and P3: Perazella score at day 1, 3 and 7 respectively

C1, C2 and C3: Chawla score at day 1, 3 and 7 respectively

IGFBP-7: insulin-like growth factor binding protein 7, KIM-1: kidney injury molecule-1, TIMP-2: tissue inhibitor of metalloproteinase 2

**Fig. 2:**
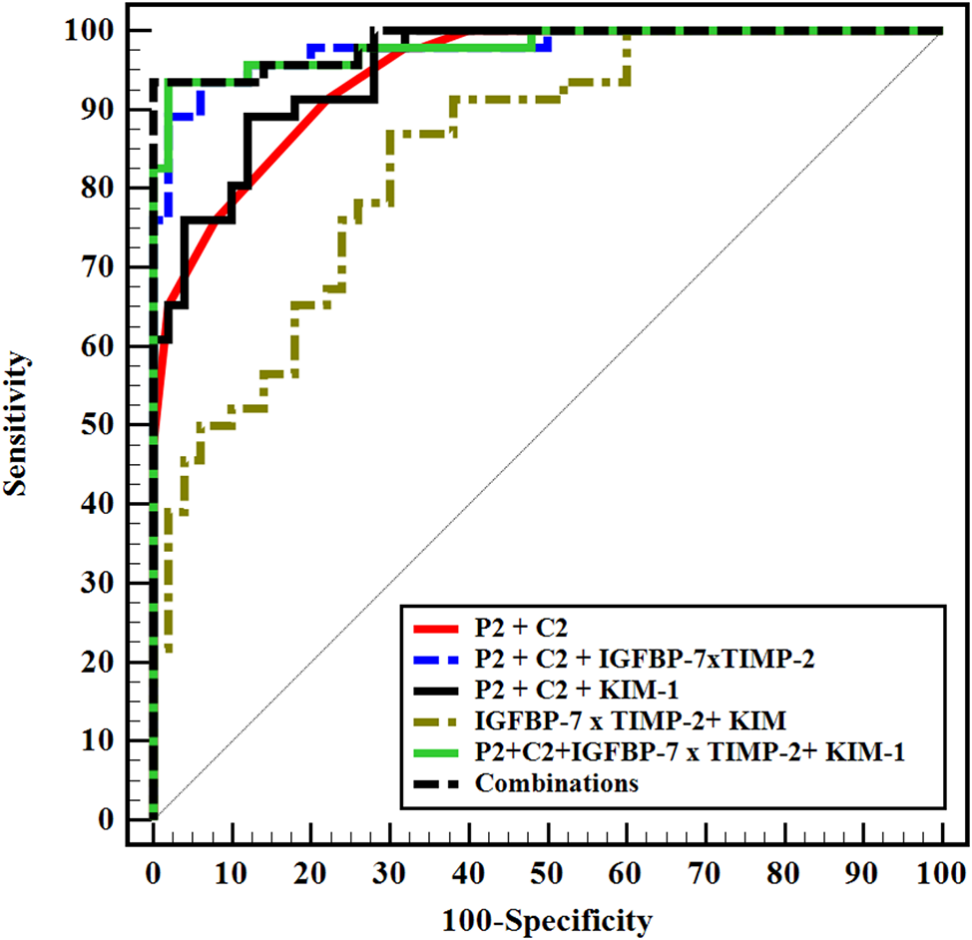
ROC curve for different combinations to predict AKI progression

Regarding prediction of AKI progression, TIMP2*IGFBP7 and KIM-1 revealed an area under the curve (AUC) of 0.837 and 0.657 respectively. Perazella score (P2 and P3) revealed an AUC of 0.893 and 0.934 respectively, while Chawla score (C2 and C3) showed an AUC of 0.920 and 0.947 respectively. Combination of sediment scores (P2 and C2) with TIMP2*IGFBP7, KIM-1, and to both markers significantly increased the AUC of biomarkers to 0.977, 0.951 and 0.979 respectively. Adding other clinical and laboratory parameters (UOP, SOFA score, urinary ACR, and procalcitonin) to sediment scores and biomarkers increased the AUC to 0.985 (Table 4; Fig. 2).

**Table 4:** Urine sediment scores, renal cell arrest and damage markers as predictors for AKI progression

	AUC	*p*	95% C.I	Cut off	Sensitivity	Specificity	PPV	NPV
**IGFBP-7 × TIMP-2**	0.837	< 0.001^*^	0.755–0.918	> 0.3	97.8	34.0	57.7	94.4
				> 2	39.1	98.0	94.7	63.6
**KIM-1**	0.657	0.008^*^	0.549–0.766	> 1295	65.22	62.0	61.2	66.0
**P1**	0.605	0.077	0.491–0.719	≥ 1	76.1	32.0	50.7	59.3
				≥ 2	37.0	84.0	68.0	59.2
				≥ 3	4.3	98.0	66.7	52.7
**P2**	0.893	< 0.001^*^	0.828–0.957	≥ 1	100.0	34.0	58.2	100.0
				≥ 2	93.5	72.0	75.4	92.3
				≥ 3	58.7	94.0	90.0	71.2
**P3**	0.934	< 0.001^*^	0.872–0.996	≥ 1	97.1	51.4	66.7	94.7
				≥ 2	94.3	82.9	84.6	93.5
				≥ 3	68.6	97.1	96.0	75.6
**C1**	0.634	0.024^*^	0.522–0.746	≥ 2	65.2	62.0	61.2	66.0
				≥ 3	2.2	98.0	50.0	52.1
**C2**	0.920	< 0.001^*^	0.867–0.974	≥ 2	97.8	66.0	72.6	97.1
				≥ 3	65.2	98.0	96.8	75.4
**C3**	0.947	< 0.001^*^	0.892–1.000	≥ 2	97.1	85.7	87.2	96.8
				≥ 3	65.7	97.1	95.8	73.9
**Combinations**								
P2 + C2	0.944	< 0.001^*^	0.905–0.984		91.3	78.0	79.2	90.7
P2 + C2 + IGFBP-7 × TIMP-2	0.977	< 0.001^*^	0.951–1.000		91.3	94.0	93.3	92.2
P2 + C2 + KIM-1	0.951	< 0.001^*^	0.914–0.988		87.0	88.0	87.0	88.0
IGFBP-7 × TIMP-2 + KIM-1	0.843	< 0.001^*^	0.767–0.919		56.5	86.0	78.8	68.3
P2 + C2 + IGFBP-7 × TIMP-2 + KIM-1	0.979	< 0.001^*^	0.954–1.000		93.5	96.0	95.6	94.1
P2 + C2 + UOP + PCT + Sofa + ACR + IGFBP-7 × TIMP-2 + KIM-1	0.985	< 0.001^*^	0.967–1.000		93.5	100.0	100.0	94.3

AUC: Area Under a Curve p value: Probability value CI: Confidence Intervals

NPV: Negative predictive value PPV: Positive predictive value

*: Statistically significant at *p* ≤ 0.05

P1, P2 and P3: Perazella score at day 1, 3 and 7 respectively

C1, C2 and C3: Chawla score at day 1, 3 and 7 respectively

ACR: albumin to creatinine ratio, IGFBP-7: insulin-like growth factor binding protein 7, KIM-1: kidney injury molecule-1, PCT: procalcitonin, SOFA: Sequential Organ Failure Assessment, TIMP-2: tissue inhibitor of metalloproteinase 2, UOP: urine output

**Fig. 3:**
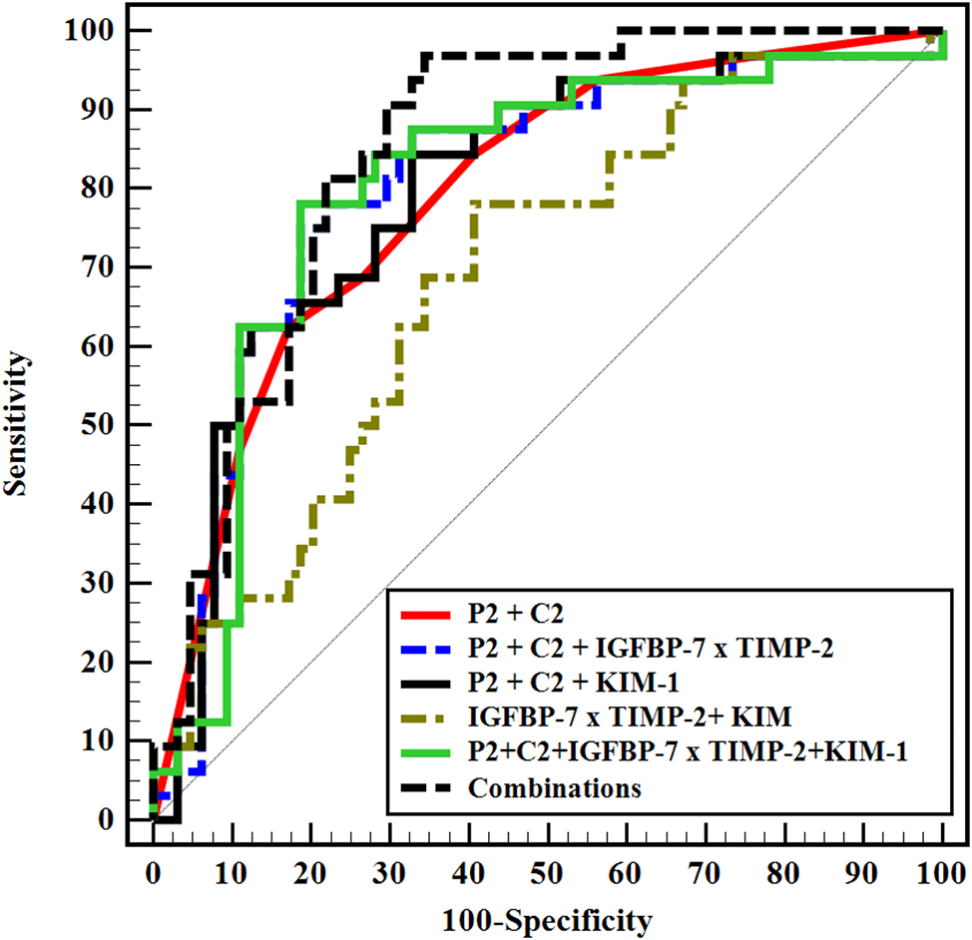
ROC curve for different combinations to predict mortality

Regarding prediction of mortality, TIMP2*IGFBP7 and KIM-1 showed an AUC of 0.679 and 0.626 respectively. Perazella score (P2 and P3) showed an AUC of 0.750 and 0.762 respectively, while Chawla score (C2 and C3) showed an AUC of 0.779 and 0.770 respectively. Adding sediment scores (P2 and C2) to TIMP2*IGFBP7, KIM-1, and to both markers significantly increased the AUC of biomarkers to 0.807, 0.796 and 0.803 respectively. Adding other clinical and laboratory parameters (UOP, SOFA score, urinary ACR, and procalcitonin) to sediment scores and biomarkers increased the AUC to 0.848 (Table 5; Fig. 3).

**Table 5:** Urine sediment scores, renal cell arrest and damage markers as a predictors for mortality

	AUC	*p*	95% C.I	Cut off	Sensitivity	Specificity	PPV	NPV
**IGFBP-7 × TIMP-2**	0.679	0.004^*^	0.564–0.794	> 0.3	87.5	21.9	35.9	77.8
				> 2	31.3	85.9	52.6	71.4
**KIM-1**	0.626	0.045^*^	0.509–0.742	> 1295	65.62	56.25	42.9	76.6
**P1**	0.599	0.116	0.472–0.725	≥ 1	75.0	29.7	34.8	70.4
				≥ 2	40.6	81.3	52.0	73.2
				≥ 3	6.3	98.4	66.7	67.7
**P2**	0.750	< 0.001^*^	0.650–0.849	≥ 1	96.9	25.0	39.2	94.1
				≥ 2	87.5	54.7	49.1	89.7
				≥ 3	53.1	79.7	56.7	77.3
**P3**	0.762	0.001^*^	0.631–0.894	≥ 1	85.7	32.7	35.3	84.2
				≥ 2	85.7	57.1	46.2	90.3
				≥ 3	71.4	79.6	60.0	86.7
**C1**	0.588	0.161	0.467–0.709	≥ 2	62.5	54.7	40.8	74.5
				≥ 3	3.1	98.4	50.0	67.0
**C2**	0.779	< 0.001^*^	0.681–0.877	≥ 2	90.6	48.4	46.8	91.2
				≥ 3	62.5	82.8	64.5	81.5
**C3**	0.770	< 0.001^*^	0.648–0.891	≥ 2	85.7	57.1	46.2	90.3
				≥ 3	66.7	79.6	58.3	84.8
**Combinations**								
P2 + C2	0.793	< 0.001^*^	0.699–0.886		62.5	82.8	64.5	81.5
P2 + C2 + IGFBP-7 × TIMP-2	0.807	< 0.001^*^	0.712–0.903		65.6	81.3	63.6	82.5
P2 + C2 + KIM-1	0.796	< 0.001^*^	0.699–0.893		62.5	82.8	64.5	81.5
IGFBP-7 × TIMP-2 + KIM-1	0.691	0.002^*^	0.582–0.801		25.0	90.6	57.1	70.7
P2 + C2 + IGFBP-7 × TIMP-2 + KIM-1	0.803	< 0.001^*^	0.705–0.900		62.5	81.3	62.5	81.3
P2 + C2 + UOP + PCT + Sofa + ACR + IGFBP-7 × TIMP-2 + KIM-1	0.848	< 0.001^*^	0.772–0.924		65.6	81.3	63.6	82.5

AUC: Area Under a Curve p value: Probability value CI: Confidence Intervals

NPV: Negative predictive value PPV: Positive predictive value

*: Statistically significant at *p* ≤ 0.05

P1, P2 and P3: Perazella score at day 1, 3 and 7 respectively

C1, C2 and C3: Chawla score at day 1, 3 and 7 respectively

ACR: albumin to creatinine ratio, IGFBP-7: insulin-like growth factor binding protein 7, KIM-1: kidney injury molecule-1, PCT: procalcitonin, SOFA: Sequential Organ Failure Assessment, TIMP-2: tissue inhibitor of metalloproteinase 2, UOP: urine output

The eGFR after 3 months showed a negative significant correlation with C2, C3, P2, and TIMP2*IGFBP7 (Table 6).

**Table 6:** Correlation between eGFR (after 3 months) with urine sediment scores, renal cell arrest and damage markers in study patients

eGFR After 3 months vs.	No.	τ_b_	*P*
IGFBP-7 × TIMP-2	64	-0.250^*^	0.004^*^
KIM-1	64	-0.017	0.843
P1	64	-0.027	0.789
P2	64	-0.229^*^	0.018^*^
P3	49	-0.141	0.206
C1	64	0.049	0.643
C2	64	-0.306^*^	0.002^*^
C3	49	-0.287^*^	0.013^*^

**τ**_**b**_: **Kendall’s tau-b**

*: Statistically significant at *p* ≤ 0.05

P1, P2 and P3: Perazella score at day 1, 3 and 7 respectively

C1, C2 and C3: Chawla score at day 1, 3 and 7 respectively

eGFR: estimated glomerular filtration rate, IGFBP-7: insulin-like growth factor binding protein 7, KIM-1: kidney injury molecule-1, TIMP-2: tissue inhibitor of metalloproteinase 2

## Discussion

AKI occurs in more than 50% of ICU patients. Costs associated with AKI are significant in this context, and prevention is challenging [16]. The most frequent cause of AKI in hospitalized patients is sepsis [17]. The 2012 KDIGO consensus criteria, which are based on changes in serum creatinine concentration or the reduction in UOP, are used to diagnose and classify AKI [5]. Despite a well-established correlation between the KDIGO-based AKI criteria and outcome, these criteria have significant and evident limitations that need to be taken into consideration [18]. Serum creatinine is influenced by numerous non-renal factors and may not increase until half of the functioning nephrons are destroyed [19]. Combining renal cell arrest with damage biomarkers and microscopic examination of U-Sed to predict the development and progression of SA-AKI was not addressed adequately. Therefore, we evaluated this concept and found that combining U-Sed examination to urinary TIMP-2 and IGFBP7, urinary KIM-1 significantly enhanced the prediction of AKI progression, and mortality compared to biomarkers alone.

In our study, progression to KDIGO AKI stage 3 occurred in (48%) of patients while (52%) of patients did not progress. AKI progressors had a significantly higher urinary TIMP2*IGFBP7 and KIM-1 measured at time of AKI diagnosis. The AUC for prediction of AKI progression for TIMP2*IGFBP7 and KIM-1 was 0.837 and 0.657 respectively, while for mortality it was 0.679 and 0.626 respectively.

Biomarkers have been proposed by numerous researchers for use in AKI evaluation, and the consensus conference of ADQI-23 offered a framework for extending the functional classification of AKI through addition of biomarkers [20]. A u[TIMP-2] × [IGFBP-7] level above 2.0 (ng/mL)^2^/1000 can indicate moderate to severe AKI with a good specificity of 95% [7]. A Cutoff of 0.3 and a cutoff 1.0 (ng/mL)^2^/1000 were also reported in the literature with higher sensitivity, but for staging, higher specificity is required [21]. According to Maize et al. [15], u(TIMP2∗IGFBP7) in early phase of septic shock identifies high-risk patients for progression to severe AKI. The likelihood of reaching KDIGO stage 3 was quadrupled by a test result more than 2.0 (ng/ml)^2^/1,000. In the Topaz study, Bihorac et al. [7] reported that uTIMP2∗IGFBP7 was an independent factor for occurrence of moderate to severe AKI within 12 h. Different cutoff values were assessed: > 0.3 (ng/ml)^2^/1,000 (showed a sensitivity of 92% and specificity of 46%) and > 2.0 (showed a sensitivity of 37% and specificity of 95%).

Regarding damage markers, KIM-1 and NGAL levels were measured in the urine and serum within 24 h of diagnosing sepsis in a Chinese study. They discovered that patients who developed AKI had greater urine and serum KIM-1 and NGAL levels than patients who did not, (AUC for prediction of AKI occurrence 0.607, 0.754, 0.768 and 0.658 respectively), while the predictive value for severe AKI (KDIGO grade ≥ 2) was lower (AUC was 0.581, 0.555, 0.727 and 0.652 respectively) and the AUC for prediction of mortality was 0.510, 0.568, 0.619 and 0.640 respectively. Combining the four biomarkers increased the AUC for AKI occurrence to 0.806 [22].

We found that combining u[TIMP-2*IGFBP-7] with uKIM-1 slightly increased the AUC to 0.843 for progression and 0.691 for mortality. Similar to our findings, one study demonstrated that when u[TIMP-2]*[IGFBP7] and uKIM-1 were combined, the AUC for AKI progression increased slightly from 0.745 to 0.752 and for AKI progression with mortality from 0.777 to 0.782 when compared to u[TIMP-2]*[IGFBP7] alone [9]. These findings imply that a more effective strategy would involve a careful combination of biomarkers than a single biomarker for AKI progression and mortality [23, 24].

In the present study, the application of serial U-Sed examination in SA-AKI was assessed and the efficacy of its addition to biomarkers. PS at the time of AKI diagnosis (P1) demonstrated no statistically significant difference between individuals with and without progression of AKI, while CS showed significant difference. By repeating urinary sediment microscopy examination on day 3 (P2 and C2) and at day 7 (P3 and C3), patients exhibiting progression of AKI demonstrated a significantly elevated score in both scores. Combining P2 and C2 had an AUC of 0.944 for predicting AKI progression.

Limited research has assessed the impact of serial U-sed examination in AKI. Varghese et al. [25] found that by repeating U-Sed microscopy, they were able to uncover around 25% of patients with ATI not identified on initial examination. Indicators of ATI on U-Sed in patients with AKI progression tend to be clustered between the 4th and 6th days after diagnosis of AKI. Likewise, few experimental studies have evaluated urinary microscopy in SA-AKI. Only one research described urine microscopy in a systematic review about urine findings in SA-AKI. While GCs, trace hematuria, RTECs, RTECCs, and pyuria were frequently reported (more than 50%), normal U-Sed was also present in number of patients [26].

In our study, we found that combining U-Sed examination with biomarkers significantly improved the predictive performance of the biomarkers alone. We found that using U-Sed scores at day 3 (P2 and C2) is better than day 1 and day 7 as PS and CS at day 1 has less predictive performance and at day 7 many patients have already progressed to AKI stage 3. Regarding AKI progression prediction, adding U-Sed (P2 and C2) to TIMP2*IGFBP7 increased the AUC from 0.837 to 0.977 and to KIM-1 increased the AUC from 0.657 to 0.951 compared to each biomarker alone. While for mortality prediction, adding U-Sed (P2 and C2) to TIMP2*IGFBP7 increased the AUC from 0.679 to 0.807 and to KIM-1 increased the AUC from 0.626 to 0.796 compared to each biomarker alone. The highest predictive performance was present when we combined all parameters (clinical and laboratory data, U-Sed, and biomarkers) which increased the AUC to 0.985 for prediction of AKI progression, and to 0.848 for mortality. In agreement with our findings, Tao X et al. [9] reported that adding clinical parameters to biomarkers improved their performance for prediction of AKI progression and mortality in patients with sepsis.

Two studies evaluated the combination of U-Sed examination and biomarkers in AKI. Elmedany SM et al. [27] reported a higher PS in AKI patients after cardiopulmonary bypass within 2 h up to second postoperative day in comparison to patients who did not develop AKI. On adding U-Sed scores to urinary NGAL and KIM-1, it was reported that the prediction performance significantly improved with high significance with an AUC of 0.906 with a 95% CI (0.812–1.001) instead of 0.801 for urinary NGAL and KIM-1. According to schinstock SA et al. [28], higher urine NGAL levels at admission were linked to a higher risk for AKI progression. As for NGAL’s ability to predict AKI, its sensitivity and specificity were only fair at 64.5% (CI: 53.3–74.3) and 64.5% (CI: 58.8–69.8), respectively. Also, they showed that the presence of one RTEC, RTECC or GC/HPF has a specificity of 91.3% but low sensitivity of 22.4%for the diagnosis of AKI, while for severe AKI stage (2 or 3), the specificity was 89.9% and sensitivity was 29.6% with increasing predictive value by adding urinary NGAL.

The strengths of our study include highlighting the great ability of a simple and cheap method like urine sed. examination to enhance the predictive power of biomarkers. In addition, we illustrated in detail the diagnostic and predictive performance of U-Sed, TIMP2*IGFBP7 and KIM-1 alone and in combination. Our study has some limitations. The sample size might be small (*n* = 96) and including more patients would have strengthen our findings. U-Sed assessment depend on trained personals which could lead to variability between operators. In anuric patients, U-Sed examination cannot be done. Additionally, other causes of AKI might be missing because renal biopsy was not done. Also, biomarkers were only assessed at the time of AKI diagnosis but not at discharge or after 3 months which does not reflect the dynamic changes of the biomarkers with progression of the condition. We utilized the CRP level and procalcitonin to reflect the level of systemic inflammation. However, more dynamic assessment of the systemic inflammatory status was needed. Lastly, assessment of myocardial function (by cardia enzymes and echocardiography) was only done when there is suspicion of myocardial injury but not routinely in all patients.

## Conclusion

In SA-AKI patients, combining serial examination of urine sediment with renal cell arrest and damage markers significantly improved the performance of the biomarkers to predict AKI progression and mortality. Additionally, combining clinical and laboratory data, U-Sed, and biomarkers provides the highest predictive performance. To validate our findings, larger randomized clinical studies are required.

## Data Availability

The corresponding author may provide the data analyzed in this study upon reasonable request.

## Abbreviations

SA-AKI: Sepsis associated acute kidney injury
TIMP-2: Tissue inhibitor of metalloproteinase 2
IGFBP7: Insulin-like growth factor binding protein 7 Subjective global assessment
KIM-1: Kidney injury molecular-1
U-Sed: Urine sediment
PS: Perazella score
CS: Chawla score
